# Labor analgesia with ropivacaine added to clonidine: a randomized clinical trial

**DOI:** 10.1590/S1516-31802008000200007

**Published:** 2008-03-06

**Authors:** Giane Nakamura, Eliana Marisa Ganem, Norma Sueli Pinheiro Módolo, Ligia Maria Suppo de Souza Rugolo, Yara Marcondes Machado Castiglia

**Keywords:** Analgesia, obstetrical, Anesthesia, epidural, Infant, newborn, Anesthetics, local, Clonidine, Analgesia obstétrica, Anestesia epidural, Neonato, Anestésicos locais, Clonidina

## Abstract

**CONTEXT AND OBJECTIVE:**

Previous studies have led to speculation that the association between ropivacaine and clonidine might be more effective than ropivacaine alone. We examined the maternal-fetal effects of two pharmacological approaches: a low dose of ropivacaine or a lower dose of ropivacaine plus clonidine for epidural analgesia during labor.

**DESIGN AND SETTING:**

Prospective study at Department of Anesthesiology, Faculdade de Medicina de Botucatu, Universidade Estadual Paulista.

**METHODS:**

Thirty-two pregnant women in American Society of Anesthesiologists physical status I and II randomly underwent epidural analgesia using 15 ml of ropivacaine 0.125% (R group) or 15 ml of ropivacaine 0.0625% plus 75 µg clonidine (RC group). Pain intensity, sensory block level, latency time, motor block intensity, duration of labor analgesia and duration of epidural analgesia were evaluated. The newborns were evaluated using Apgar scores and the Amiel-Tison method (neurological and adaptive capacity score).

**RESULTS:**

There were no statistically significant differences between the groups regarding pain score, sensory block level, duration of epidural analgesia or Apgar score. The latency time, duration of labor analgesia and motor block were R group < RC group. The half-hour and two-hour neurological and adaptive capacity scores were higher in the R group. All of the R group newborns and 75% of the RC group newborns were found to be neurologically healthy at the 24-hour examination.

**CONCLUSION:**

Both low-dose ropivacaine and a lower dose plus clonidine relieved maternal pain during obstetric labor. Newborns of mothers who received only ropivacaine showed better neurological and adaptive capacity scores.

**CLINICAL TRIAL REGISTRATION NUMBER:**

NCT00626977.

## RESUMO

**CONTEXTO E OBJETIVO:**

A associação entre ropivacaína e clonidina agiria menos que a ropivacaína isolada na mãe e no feto? Foram pesquisados os efeitos materno-fetais de duas técnicas farmacológicas: pequena dose de ropivacaína ou dose menor de ropivacaína mais clonidina na analgesia peridural para parto.

**TIPO DE ESTUDO E LOCAL:**

Estudo prospectivo, Departamento de Anestesiologia, Faculdade de Medicina de Botucatu, Universidade Estadual Paulista.

**MÉTODOS:**

Trinta e duas parturientes, estado físico de acordo com a American Society of Anesthesiologists I e II, foram aleatoriamente submetidas à analgesia peridural com 15 ml de ropivacaína 0,125% (grupo R) ou 15 ml de ropivacaína 0,0625% mais clonidina, 75 µg (grupo RC). Foram avaliados: intensidade da dor, nível do bloqueio sensitivo, latência, intensidade do bloqueio motor, duração da analgesia de parto e da analgesia peridural. Os neonatos foram avaliados pelo Apgar e método de Amiel-Tison (capacidade neurológica e adaptativa).

**RESULTADOS:**

Não houve diferenças significativas entre grupos para dor, nível de bloqueio sensitivo, duração da analgesia peridural e Apgar. Para latência, duração da analgesia de parto e bloqueio motor, grupo R < grupo RC. O escore da capacidade neurológica e adaptativa de meia e duas horas foi maior para o grupo R. Cem por cento dos neonatos do grupo R e 75% dos do grupo RC estavam neurologicamente saudáveis ao exame de 24 horas.

**CONCLUSÃO:**

Pequena dose de ropivacaína e dose menor mais clonidina aliviaram a dor materna durante o parto. Neonatos de mães que receberam apenas ropivacaína mostraram melhores escores da capacidade neurológica e adaptativa.

## INTRODUCTION

Ropivacaine is less toxic to the cardiovascular system than bupivacaine^1^ and is more selective for sensory fibers, thus determining lower motor blockade.^2^ We have found^3^ better Apgar scores and neurological and adaptive capacity scores (Amiel-Tison method)^4^ when using a low concentration (0.125%) and low dose of epidural ropivacaine for labor analgesia and anesthesia.

Clonidine, combined with a local anesthetic, increases the quality and duration of epidural analgesia through a mechanism that involves spinal α_2_-adrenoreceptors,^5^ without interfering with proprioception and without causing the respiratory depression, nausea or pruritus that opioids do. However, α_2_-receptor agonist induces hypotension by inhibiting the sympathetic tone. Oral use of clonidine has been described as a means for treating hypertension in pregnant women, without adverse effects on the fetus.^6^ Landau et al.^7^ demonstrated a dose-sparing effect of clonidine (75 µg) when added to different doses of ropivacaine (0.1% and 0.2%) in early active labor.

Reviewing the use of clonidine as a neuraxial adjuvant drug, Roelants^8^ concluded that the most interesting aspect of this is its epidural use. Epidural clonidine would produce prolonged analgesia from local anesthetics and opioids and would allow a local anesthetic sparing effect. The optimal epidural dose would lie between 60 µg to 75 µg: a dose lower than 60 µg is ineffective, whereas a dose larger than 100 µg induces sedation and hypotension.

The prospective and randomized study we previously conducted compared 0.125% bupivacaine, ropivacaine and levobupivacaine for labor epidural analgesia and evaluated the parameters of mothers and newborns.3 Ropivacaine 0.125% was adequate for analgesia and showed better neurological and adaptive capacity scores for the newborns. The question that arises from this is whether, if ropivacaine were used in combination with clonidine for analgesia, any synergism allowing its dose to be decreased would occur. A further question to be addressed is whether this association would act less than ropivacaine alone on the mother and newborn.

## OBJECTIVE

The aim of the present study was to examine the efficacy of two pharmacological approaches for pain relief during labor: 0.125% ropivacaine alone or 0.0625% ropivacaine added to 75 µg clonidine, both by epidural administration. The effect of the drugs on the mother and newborn was also determined.

## METHODS

The study was approved by the local Clinical Research Ethics Committee and written informed consent was obtained from the patients before the onset of labor analgesia. All patients had a singleton healthy full-term pregnancy and were classified as American Society of Anesthesiologists physical status I or II. Patients who had received opioids or presented a history of hypersensitivity to local anesthetic or to clonidine, or whose fetus showed signs of possible intrauterine suffering (non-reassuring heart rate tracing) or neurological deficit, were excluded from the study.

In this randomized clinical trial (conducted by means of drawing lots), thirty-two women who had requested epidural analgesia for labor were randomly allocated to two groups (n = 16 each), to receive 15 ml of either 0.125% ropivacaine (18.75 mg) (R group) or 0.0625% ropivacaine (9.375 mg) plus 75 µg clonidine (RC group).

In cases in which a second dose of local anesthetic was necessary for labor analgesia in response to patient demand, rather than because of inadequate block, a bolus of 6 ml of ropivacaine was administered at 0.125% (7.5 mg) concentration to the women in the R group or 0.0625% (3.75 mg) concentration without clonidine to the women in the RC group.

After obstetrical indication of labor analgesia, the cervical dilatation and intensity of pain were recorded using a numerical verbal scale (zero = no pain; 10 = worst pain). The maternal parameters monitored included arterial blood pressure by a noninvasive method, heart rate and oxygen saturation by pulse oximetry. An intravenous infusion of 500 ml of lactated Ringer’s solution was administered before the epidural injection. With the patient in a sitting position, an epidural catheter was inserted into the L3-L4 vertebral interspace using the loss of resistance technique; 2-3 cm of catheter were introduced in the epidural space. With the patient in the supine position again, a test dose of local anesthetic (3 ml of 1% lidocaine and 1:200,000 epinephrine) was administered through the catheter and the study drugs were then administered through it.

The following parameters were then assessed: 1) pain using a numerical verbal scale (before the epidural analgesia and after implementing the sensory blockade); 2) level of the sensory blockade, determined up to 20 min after local anesthetic administration; 3) latency time (time between the drug administration and the achievement of the highest sensory blockade level), determined at one-minute intervals; and 4) degree of motor blockade, determined by testing the rectus abdominis muscles^9^ 20 minutes after implementing the sensory blockade, such that if the parturient still presented 100% power, she was able to rise from a supine to a sitting position with hands clasped behind her head; 80%, she succeeded only by extending her arms; 60%, she could only lift her scapulae from the bed; 40%, she could only lift her shoulders; and 20%, she only felt an increase in tension during the effort. The duration of labor analgesia was also assessed and was defined as the time between first local anesthetic administration and local anesthetic administration for the delivery (for episiotomy or cesarean section). The duration of epidural analgesia was defined as the time from the beginning of analgesia to childbirth.

For vaginal delivery, 8 ml of 0.5% ropivacaine were administered, while for cesarean section, 20 ml of 0.5% ropivacaine were administered.

The type of delivery and the neonatal Apgar scores at 1, 5 and 10 min were recorded. The gestational age (in days) was determined by Capurro’s method.^10^ In the nursery, the newborns were again evaluated, always by the same trained anesthesiologist in a well-lit, heated environment without much noise, at 30 minutes, two hours and 24 hours after birth. The evaluations in the nursery were done using the Amiel-Tison method,^4^ in which scores from evaluating the adaptive capacity, passive tone, active tone, primary reflex and overall vision were summed to give the final neurological and adaptive capacity score. A total score ≥ 35 is indicative of a neurologically vigorous newborn, according to the method. The anesthesiologist who evaluated the newborns was blinded to the maternal analgesic regimen.

Maternal hypotension was defined as systolic arterial pressure < 100 mmHg. It was treated by increasing the rate of the intravenous infusion, positioning the patient on her left side and, if necessary, administering ephedrine.

### Statistical analysis

The data were analyzed statistically. For the categorical variables, the proportions of the variances in the two groups were compared using the chi-squared test with calculation of the χ2 statistic and p value, or using Fisher’s exact test. For semiquantitative variables, the groups were compared using the Mann-Whitney test. To compare the time points within each group, the Friedman test for dependent variables was used, with calculation of the χ2 statistic and the p value complemented with the contrasts between point sums between time pairs. For quantitative variables (measurements), the groups were compared using Student’s t test for independent samples. For all statistical analyses, the level of significance was p < 0.05.

The number of subjects in each group was determined to detect (with a power of 0.8 and p < 0.05) a difference of eight points in the neurological and adaptive capacity scores between the two groups.

## RESULTS

In each group, there were 16 patients and newborns. The demographic data did not differ between the two groups (Table 1). There was no significant difference in the pain before epidural blockade and after the induction of labor analgesia between the groups. The median cervical dilatation was greater in the R group at the time of inducing epidural analgesia (8 cm, versus 7 cm in the RC group) (Table 2).

**Table 1:** Maternal data: 32 women receiving two different regimens of analgesia during labor

	Ropivacaine (n = 16)	Ropivacaine + Clonidine(n = 16)
Age (years)	19.90±3.57	21.40±8.10
Height (cm)	160.60±5.50	159.10±7.75
Weight (kg)	74.88±10.46	71.06±9.55
Body mass index	29.63±4.62	28.08±3.35
ASA (I/II)	14/2	12/4

*ASA = American Society of Anesthesiologists physical status; Data presented are mean ± standard deviation, % or n; no significant differences between groups (p > 0.05).*

**Table 2:** Characteristics of delivery and analgesia: 32 women receiving analgesia during labor

	Ropivacaine(n = 16)	Ropivacaine + clonidine(n = 16)
Numerical verbal scale at epidural implementation*2	10	10
Numerical verbal scale after analgesia*2	0	1
Initial cervical dilation (cm)*2,‡	8	7
Latency time (min)†,‡	8.10 ± 3.42	12.1 ± 6.60
Sensory blockade level*2	T 6	T 6
Rectus abdominis testing (%)*2,‡	80	60
Duration of labor analgesia (min)†,‡	42.40 ± 30.70	79.90 ± 48.54
Duration of epidural analgesia (min)†	71.70 ± 46.36	101.10 ± 54.49
Vaginal delivery (%)	69	63
Cesarean section (%)	31	37

* *Value reported as median;*.

† *Value reported as mean ±standard deviation;*

‡ *p < 0.05.*

Latency time, motor blockade and duration of labor analgesia differed significantly, such that the RC group was greater than the R group (p < 0.05). There were no differences in the level of sensory blockade or duration of epidural analgesia (Table 2). Supplementary analgesia (bolus dose) was necessary in both the R and RC groups (one second dose), but the differences in the number of mothers who required this additional dose (one in the R group and four in the RC group) and the time elapsed between the first and the second dose were not significant between the groups.

Maternal hypotension occurred in one patient in the R group and in two patients in the RC group during the resolution of delivery (without any significant difference). All of these patients were successfully treated with ephedrine.

The newborns did not differ in weight or gestational age (Table 3). The Apgar scores were the same in both groups at the first minute and at 10 minutes, but were greater in the R group than in the RC group at 5 minutes (p < 0.05) (Table 3).

**Table 3:** Characteristics of newborns from mothers receiving analgesia during labor

	Ropivacaine(n = 16)	Ropivacaine + clonidine (n = 16)
Weight* **(g)**	3431.56 ± 578.75	3351.87 ± 487.41
Capurro* **(days)**	278.94 ± 7.78	278.75 ± 11.84
**1st min Apgar score** †	8	8
**5th min Apgar score**†,‡	10	9
**10th min Apgar score** †	10	10
**Adaptive capacity**† **(score)**		
30 min‡	7	5
2 h‡	8.5	8
24 h	10	10
**Passive tone**† **(score)**		
30 min‡	6.5	3
2 h	7	7
24 h	8	8
**Active tone**† **(score)**
30 min‡	6.5	4
2 h‡	7	6
24 h	9	8
**Primary reflex**† **(score)**		
30 min	4	4
2 h‡	5	4
24 h	5	5
**Overall vision**† **(score)**		
30 min	6	4
2 h	6	5.5
24 h	6	6
**Neurological adaptive capacity** †		
30 min‡	29.5	21
2 h‡	33	29
24 h	37.5	36

* *Value reported as mean ± standard deviation;*.

† *Value reported as median;*.

‡ *p < 0.05.*

The median score for each item in the Amiel-Tison method is presented in Table 3. It can be seen that the median adaptive capacity of the newborns during the first 30 minutes and at two hours was greater in the R group than in the RC group (p < 0.05). No significant difference was observed between groups at the 24-hour examination. The passive tone in the R group was greater than in the RC group (p < 0.05) at the 30-minute time point, but at two hours and 24 hours, the groups did not differ. At the 30-minute and two-hour examinations, the active tone was significantly greater in the R group than in the RC group (p < 0.05), but at 24 hours, the results were not considered different. At the 30-minute and 24-hour examinations, the results from the R and RC groups were the same for primary reflex, but at the two-hour examination, the reflex was greater in the R group (p < 0.05). At the three times analyzed, the two groups were equal in terms of overall vision. At the 30-minute and two-hour examinations, the neurological and adaptive capacity score was greater in the R group than in the RC group (p < 0.05), but at the 24-hour examination, the results were the same for the two groups. At the two-hour (p < 0.05) and 24-hour examinations, greater occurrence of neurological and adaptive capacity scores greater than 35 was observed in the R group than in the RC group (Table 4).

**Table 4:** Percent of neurological and adaptive capacity score ≥ 35 among newborns from 32 mothers receiving analgesia during labor

	Ropivacaine(n = 16)	Ropivacaine + clonidine(n = 16)
2 h*	25	0
24 h	100	75

* *p < 0.05.*

## DISCUSSION

To explain the interaction of clonidine with local anesthetic in regional blockades, the following mechanisms are suggested: 1) direct action blocking the conduction of stimuli in Aδ and C fibers, increasing the conductance to K^+^ in isolated neurons and intensifying the blockade of local anesthetic conduction^11^; 2) indirect action reducing local anesthetic absorption by means of a vasoconstricting effect mediated by postsynaptic α_2_-receptors located in the smooth muscles of epidural vessels.^12^

There was a significant difference in cervical dilation at the time of analgesia, such that the R group patients had a more dilated cervix. The R group presented a significantly shorter duration of labor analgesia than RC group did, but an equal duration of epidural analgesia. Thus, the periods of time during which the newborns of the mothers in the two groups remained under the effect of the agents used did not differ significantly. In a study by Cigarini et al.,^13^ there was an increase in the duration of labor among the patients receiving clonidine and local anesthetic, compared with the control receiving only the same dose of local anesthetic.

The test dose used in the present study does not seem to have masked any differences between the two groups. Due to its low content of local anesthetic, it would not have provided labor pain analgesia, yet it was safe for both the mother and the fetus. Abraham et al.^14^ observed sensory block at S_2_ level in parturients receiving 2 ml of 1.5% intrathecal lidocaine (30 mg). This dose, which is lower in epidural than in intrathecal administration, would contribute towards the mother’s sensory block and the newborn’s neurobehavior.

The latency time was shorter in the group that received only local anesthetic. This result was the same as found in a clinical study comparing labor analgesia between patients receiving an equal volume of 0.125% bupivacaine alone or combined with 120 µg clonidine.^15^

When only the local anesthetic was administered, the patients were able to move better. This may also have contributed towards longer duration of analgesia in the RC group. Some authors13 have observed that clonidine contributes towards greater motor blockade.

There was no significant difference in pain intensity after starting the labor analgesia between the groups. When comparing the labor analgesia obtained with bupivacaine added to 75 µg clonidine with that obtained for the control group, other authors have observed better analgesia in the clonidine group.^13,15^

The need for additional local anesthetic among the patients in the present study demonstrated that clonidine allowed the duration of analgesia in the RC group to be equal to that of the R group. Cigarini et al.^13^ demonstrated that patients who received only bupivacaine required additional local anesthetic for labor analgesia within a shorter period of time than did women who received clonidine. However, these authors used equal doses of local anesthetic for the two groups studied.

When clonidine is combined with epidural local anesthetics, hypotension is no more marked than after epidural administration of a plain solution of local anesthetic.^16^ The hypotension induced by clonidine results from the inhibition of sympathetic tone. This effect is also caused by the local anesthetic, and would be related to the level of the epidural space at which the drug is administered.^17^

Several types of neonatal depression or lesion are readily apparent and easily detected by conventional neurological examination. However, slight depression or lesion is not easily detected in neonates. Infants with a high Apgar score may have mild neurological signs of depression due to drugs (i.e., mild hypotonia, lower primary reflex responses etc). Perinatal asphyxia and mild delivery trauma may result in small differences in the tone of neck extensor and flexor muscles and hypotonia of the upper limbs.^18,19^ The neurological and adaptive capacity score was developed to differentiate drug-induced depression from depression due to asphyxia, delivery trauma or neurological disease in neonates.^4^

In a review,^20^ some criticisms of the Amiel-Tison test were made, relating to variability of the maternal anthropometric data, exclusion/inclusion factors for neonates and gestational age. In the present investigation, we did not study neonates who already presented factors that might interfere with the examination prior to the indication of analgesia, and the two groups did not differ significantly in terms of Capurro index or birth weight. The samples in the two groups studied were homogeneous and consisted of young non-obese patients, thus invalidating the criticisms cited above.

Halpern et al.^21^ also assessed the reliability of the Amiel-Tison method, with two teams of observers trained to perform the test on healthy, full-term neonates delivered in the vertex presentation. They concluded that neurological and adaptive capacity scores presented poor reliability, both in simultaneous testing and in test-retest situations. Some items of the test are objective (tone) and the authors used one examiner to perform and one examiner to observe. They also chose an interval of time short enough to reduce the likelihood of the infant’s neurobehavior changing but on the other hand, it was also short enough for them to recover from the initial examination (30 minutes).

One study demonstrated that ropivacaine crosses the placenta after epidural administration and its non-protein-bound quantities in the maternal and umbilical veins at delivery were higher than those reported for bupivacaine.^22^ However, infants born to women who received 0.25% ropivacaine or bupivacaine for labor analgesia evolved with the same neurological and adaptive scores.^23^ In contrast, a study by Gaiser et al.^24^ detected a larger number of newborns with a neurological and adaptive score ≥ 35 after 0.25% ropivacaine than after 0.25% bupivacaine, at examinations 15 minutes (89% versus 81%) and 2 hours (95% versus 86%) after birth.

Systemically administered clonidine easily crosses the placental barrier. The ratio between the fetal and maternal plasma concentration is approximately 0.9,13,19 since the drug is metabolized less by the fetus and is slowly eliminated by the fetus and neonate.^25^ Some studies13,15 have reported that newborns delivered by mothers who had received clonidine for labor analgesia did not differ from the control group in terms of Apgar score.

Thus, when clonidine and ropivacaine crossed the placenta, they interfered with neurological and adaptive factors separately. The ropivacaine group was always less compromised at the 30-minute and two-hour examinations. Taking all of these factors together, the neurological and adaptive score calculations showed higher scores for the R group at the 30-minute and two-hour examinations, as demonstrated by statistical analysis. The newborns in the RC group were born with lower scores and evolved over time until they reached the same score as observed in the R group at 24 hours. This may have been due to interference by clonidine. The drug would cross the placental barrier and induce sedation in the newborns for a few hours following delivery, with progressive recovery thereafter.

However, Claes et al.^26^ not only determined Apgar scores for newborn infants delivered by mothers who had received epidural clonidine for labor analgesia, but also analyzed the two and 24-hour neurological and adaptive scores and found no differences between their groups.

On the other hand, studying intrathecal clonidine (30 µg) for labor pain relief, Missant et al.^27^ concluded that this drug prolonged spinal analgesia with ropivacaine (3 mg) and sufentanil (1.5 µg) but at the expense of maternal hypotension, worse fetal wellbeing and worse neonatal umbilical artery pH. They did not recommend routine administration of spinal clonidine in the way it was used in their study.

## CONCLUSION

The present study showed that both a low dose of ropivacaine and a lower dose of ropivacaine added to clonidine were effective for labor pain relief. However, ropivacaine with clonidine interfered more with the neurological and adaptive capacity of the newborn.
